# The Association of Headache and Physical Activity in Times of the COVID‐19 Pandemic: A Prospective Cohort Study

**DOI:** 10.1002/brb3.71490

**Published:** 2026-07-06

**Authors:** Anne‐Mari Torgersen Dalgeir, Ida Henriette Caspersen, Edoardo Caronna, Per Magnus, Lill Trogstad, Marte‐Helene Bjørk

**Affiliations:** ^1^ Department of Physiotherapy Haukeland University Hospital Bergen Norway; ^2^ NorHead, Norwegian Centre for Headache Research Norwegian University of Science and Technology Trondheim Norway; ^3^ Centre for Fertility and Health Norwegian Institute of Public Health Oslo Norway; ^4^ Headache Unit, Neurology Department Vall D'hebron University Hospital Barcelona Spain; ^5^ Headache Research Group, Departament de Medicina, Vall d'Hebron Institute of Research Universitat Autonoma de Barcelona Barcelona Spain; ^6^ Department of Method Development and Analysis Norwegian Institute of Public Health Oslo Norway; ^7^ Department of Clinical Medicine University of Bergen Bergen Norway; ^8^ Department of Neurology Haukeland University Hospital Bergen Norway

**Keywords:** COVID‐19 pandemic, headache, MoBa, physical activity, SARS‐CoV‐2

## Abstract

**Background:**

Prospective studies of the risk of headache with low levels of physical activity are limited. Pandemic‐related lifestyle changes and the potential headache risk associated with Coronavirus Disease (COVID‐19) and SARS‐CoV‐2 vaccination may complicate this association. Our study aims to investigate the potential risk of new bothersome headache in relation to physical activity and explore the impact of COVID‐19 and SARS‐CoV‐2 vaccines on this association.

**Methods:**

This prospective cohort study utilized questionnaires from the Norwegian Mother, Father, and Child Cohort Study (MoBa). Logistic regression analyses were conducted to estimate the adjusted odds ratio (aOR) for new‐onset headache according to physical activity levels. Models were adjusted for gender, age, body mass index, education level, smoking, alcohol intake, anxiety/depression, and COVID‐19 and SARS‐CoV‐2 vaccination prior to February 2022. The potential impact of COVID‐19 disease was investigated in a stratified analysis.

**Results:**

Both low level of physical activity and inactivity were significantly associated with new bothersome headache when compared to moderate to high activity levels (low activity: aOR 1.22, 95% CI 1.13 to 1.30; inactive: aOR 1.26, 95% CI 1.05 to 1.51). The corresponding adjusted absolute risk differences were 1.9% (95% CI 1.2 to 2.6) and 2.3% (95% CI 0.4 to 4.3), respectively. Findings persisted across COVID‐19 status strata, without significant interactions.

**Conclusion:**

Our findings underscore the potential role of physical activity in mitigating new bothersome headache also for headache associated with COVID‐19. Encouraging regular physical activity may serve as a preventive measure for headache in a pandemic setting.

## 1 Introduction

Headache disorders are among the most prevalent and disabling neurological conditions worldwide (James et al. 2018), with both environmental and lifestyle factors contributing to their onset and severity (Amiri et al. 2022; Holroyd et al. 2000). Although regular physical activity is associated with improved overall health and less pain, the interplay with headache disorders remains complex and not yet fully understood. Exercise has been shown to reduce several headache symptoms, and inactivity seems to be a risk factor for developing headache (Amin et al. 2018). On the other hand, some individuals describe exacerbation of headache symptoms during or after physical activity (Amin et al. 2018).

The Coronavirus Disease (COVID‐19) pandemic introduced additional factors that may have influenced headache occurrence, including SARS‐CoV‐2 infection and vaccination, both of which have been linked to headache as a common symptom (Caronna et al. 2023; Fernández‐De‐Las‐Peñas et al. 2021; Göbel et al. 2021). However, whether these factors modify the association between physical activity and headache remains unknown.

Existing evidence indicates a potential link between physical activity and COVID‐19, as inactive individuals had an increased risk for severe disease and more hospitalizations (Fernández‐De‐Las‐Peñas et al. 2021). Another study identified a negative association between physical activity levels and post‐COVID‐19 symptoms (Jimeno‐Almazán et al. 2022). Despite these findings, little is known about how physical activity interacts with COVID‐19‐related headache and whether infection or vaccination alters the potential risk of headache associated with physical activity.

To investigate these relationships, we aimed to estimate the association between physical activity levels and the risk of developing bothersome headaches, using a large population‐based cohort. Additionally, we explored whether SARS‐CoV‐2 infection and vaccination modified this association, a question of particular relevance for headache prevention in the context of future pandemics.

## 2 Methods

### 2.1 Study Design and Participants

The Norwegian Mother, Father, and Child Cohort Study (MoBa), conducted by the Norwegian Institute of Public Health, is a population‐based pregnancy cohort study that recruited participants from 1999 to 2008. With a participation rate of 41%, the cohort comprises 95,000 mothers, 75,000 fathers and their children recruited from all parts of Norway. Invitations to participate were sent to pregnant women and their partners by mail. Every participant has provided a written informed consent to participate in the study and for long‐term data storage (Magnus et al. 2016).

MoBa aims to investigate the causes of diseases through longitudinal follow‐ups via electronic questionnaires and medical registers. Since March 2020, approximately 170,000 active adult cohort participants from MoBa were invited to complete electronic questionnaires every 14 days regarding COVID‐19‐related symptoms. This sub‐cohort aimed to monitor COVID‐19 in Norway, seeking to understand why some individuals become infected and potentially seriously ill (Folkehelseinstituttet 2023).

**FIGURE 1:**
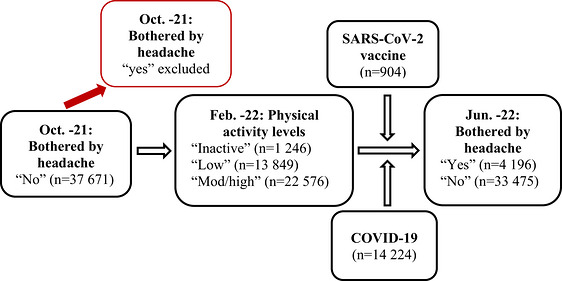
Study flow diagram showing participant selection and number of observations at each step. The study is based on three MoBa COVID‐19 questionnaires: October 2021 (headache status for eligibility), February 2022 (physical activity, exposure), and June 2022 (new bothersome headache, outcome). SARS‐CoV‐2 vaccination and infection data were obtained from national health registries, with supplementary self‐reported infection data collected through questionnaires.

The current study is based on the “Using MoBa to Understand the COVID‐19 Pandemic” sub‐cohort and includes mothers and fathers who responded to relevant questionnaires on headache and physical activity between October 2021 and June 2022. October 2021 marks the baseline for this study, as it was the first time this COVID‐19 cohort received a questionnaire addressing headache. Participants reporting headache during the year prior to baseline were excluded. The outcome therefore reflects new‐onset headache during follow‐up. Data on physical activity levels (exposure) were obtained from a questionnaire distributed in February 2022, and data on new bothersome headache (outcome) were obtained from a questionnaire distributed in June 2022. Participants who did not respond to these questionnaires were excluded. The process of participant selection and timing of data collection is illustrated in Figure 1.

### 2.2 Variables

#### 2.2.1 Outcome Measures

Outcome data on headache status was determined through responses to a specific questionnaire item sent in June 2022, assessing whether participants had experienced bothersome headaches within the last year dichotomized into “yes” (bothered by headache within the last year) or “no” (not bothered by headache within the last year). This questionnaire item has previously been validated against telephone interviews, demonstrating moderate agreement in identifying bothersome headache (Hagen et al. 2000).

The outcome reflects new‐onset bothersome headache during follow‐up, as participants reporting headache within the year prior to baseline (October 2021) were excluded from the analysis.

### 2.3 Exposure Variables

Engagement in physical activity was evaluated through two specific questionnaire items distributed to participants in February 2022. The first question asked about frequency of engaging in physical activity during leisure time, with 5 response options ranging from “never” to “almost every day.” Those who reported any activity were then asked about the intensity of their activity, ranging from low, medium, and high intensity. Every response option came with a description of each intensity level, for example, “I take it easy without breaking into a sweat or losing my breath.” The two inquiries measuring physical activity have been validated and showed correlation with peak oxygen uptake measures and the validated questionnaire “International physical activity questionnaire” (Kurtze et al. 2007; Nes et al. 2011).

Responses were subsequently categorized into three levels in accordance with the World Health Organization's (WHO) recommendations of physical activity (World Health Organization 2020), classifying individuals as inactive, low physically active or moderate/high physically active. Persons answering that they never engaged in physical activity were categorized as inactive. Participants who engaged in physical activities less than once a week or reported activities to be of low intensity were categorized as low physical active. Finally, persons considered meeting the recommendations on physical activity set by WHO (World Health Organization 2020), were categorized as moderate/high physically active. This category consisted of persons being physical active of moderate or high intensity 2–3 times a week or more. This consolidation of data aimed to provide a more comprehensive representation of physical activity compared to using the two questions separate.

### 2.4 Covariates

We adjusted for covariates implicated in the association between physical activity and headache in previous literature such as age, sex, body mass index (BMI), education level, anxiety/depression, alcohol consumption, and smoking status. All variables were based on questionnaire responses collected prior to October 2021, including data from the original MoBa study. For items that were measured at multiple time points during the MoBa study and the COVID‐19 cohort, the most recent response was used. If a participant had not responded to the most recent item, earlier available responses were used to reduce missing data. Age was treated as a continuous variable. BMI, calculated as weight (kg) divided by height (m^2^), was also treated as a continuous variable. Education level and smoking status were retained as categorical variables, following their original response formats. Alcohol intake was originally categorized into seven levels based on the response format and was recategorized into four categories for the analyses (never, less than weekly, 1–3 times per week, and ≥4 times per week). Symptoms of anxiety and depression were assessed utilizing the 5‐item version of the standardized questionnaire Hopkins Symptom Checklist (HSCL‐5). Responses were dichotomized based on the established cut‐off score of ≥2.0 (Strand et al. 2003).

### 2.5 SARS‐CoV‐2 Infection and Vaccination

SARS‐CoV‐2 infection and vaccination status were primarily obtained from the mandatory Norwegian registries that contain such information from all inhabitants of Norway. SARS‐CoV‐2 vaccination information is continuously registered in the National Immunization Registry SYSVAK, while SARS‐CoV‐2 infection is registered in the Norwegian Surveillance System for Communicable Diseases, MSIS. Additionally, information on self‐reported positive COVID‐19 tests, confirmed either by the polymerase chain reaction test or antigen‐based rapid tests were collected from inquires included in every questionnaire.

Information on SARS‐CoV‐2 infection and vaccination was categorized according to whether events occurred before or after completion of the physical activity questionnaire in February 2022. Events occurring prior to this time point were treated as potential covariates and adjusted for in the primary analyses investigating the association between physical activity and headache.

Events occurring after February 2022 but before the headache outcome assessment in June 2022 were included in sub‐analyses examining whether SARS‐CoV‐2 infection modified the association between physical activity and new bothersome headache. Additionally, a supplementary variable was constructed for descriptive purposes capturing infection and vaccination status prior to October 2021.

### 2.6 Statistical Methods

Descriptive statistics were analyzed and reported as means with standard deviations (SD) for continuous variables, and number of observations and percentages for categorical variables. Unadjusted and adjusted logistic regression analyses were used to investigate the association between physical activity and new bothersome headache, with results presented as odds ratios (OR) with 95% confidence intervals (CI). The impact of COVID‐19 and SARS‐CoV‐2 vaccination between February 2022 and June 2022 was investigated by stratified analysis and in multivariable analyses including an interaction term (physical activity × COVID‐19) to investigate the possible impact on the relationship between physical activity and headache. In addition, absolute risks (AR) and absolute risk differences (ARD) were estimated from predictive margins of the logistic regression models, both for the overall sample and within the COVID‐19 stratified analyses.

The proportion of subjects with missing information in any variable was low (6%), and analyses were run based on complete cases without additional imputation. All analyses were conducted with Stata/MP version 17.0 (StataCorp, College Station, Texas, USA).

### 2.7 Ethics

The establishment of MoBa and initial data collection was based on a license from the Norwegian Data Protection Agency and approval from The Regional Committees for Medical and Health Research Ethics. The MoBa cohort is now based on regulations related to the Norwegian Health Registry Act. The current sub‐study was approved by The Regional Committee for Medical and Health Research Ethics, Southeast Norway C, no. 127708.

## 3 Results

### 3.1 Participants

**FIGURE 2:**
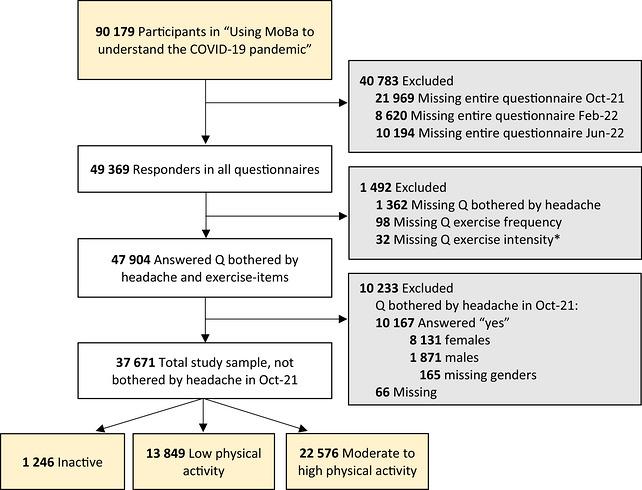
Flowchart. *Missing in intensity and answers ≥2–3 times a week in frequency, the rest of missing in intensity were analyzed.

A total of 90,179 participants were included in the current study. We excluded in total 42,275 individuals due to missing entire questionnaires or inquiries of outcome‐ and exposure data. After exclusion of persons who reported to be bothered by headache within the last year at baseline (in October 2021), we ended with a total study sample of 37,671 individuals (Figure 2).

### 3.2 Descriptive Data

There was a slight predominance of men (*n* = 16,028, 57%) in the final study sample, down from a predominance of 62% women before excluding participants with pre‐existing bothersome headache. The mean age was 50 years (SD 5.2). Two thirds (*n* = 24,407, 67%) had a college education. A detailed description of the participants is presented in Table 1. Only 2% of the participants had been infected by COVID‐19 prior to October 2021, while 98% had been vaccinated in the same period. By February 2022, a total of 20% of the sample had been infected by COVID‐19.

**TABLE 1:** Descriptive statistics of study sample characteristics.

	Inactive (*N* = 1246)	Low physical activity (*N* = 13,849)	Moderate to high physical activity (*N* = 22,576)	Total (*N* = 37,671)
Age				
Mean (SD)	49.9 (5.75)	50.1 (5.39)	49.8 (5.10)	49.9 (5.22)
Gender, male, *N* (%)	678 (56)	5601 (59)	9749 (56)	16,028 (57)
BMI				
Mean (SD)	28.4 (5.60)	27.0 (4.73)	25.6 (3.95)	26.2 (4.38)
Education level, *N* (%)				
>4 years college ≤4 years college High school <High school	172 (14) 321 (27) 522 (44) 174 (15)	3155 (24) 5146 (38) 4242 (32) 830 (6)	6739 (31) 8774 (40) 5556 (23) 841 (4)	10,066 (28) 14,341 (39) 10,320 (28) 1845 (5)
Smoking, *N* (%)				
No Sometimes Daily	1015 (82) 58 (5) 172 (14)	12,477 (90) 535 (4) 822 (6)	21,388 (95) 676 (3) 482 (2)	34,880 (93) 1269 (3) 1476 (4)
Alcohol, *N* (%)				
Never Less than weekly 1–3 times per week ≤4 times per week	205 (16) 580 (47) 377 (30) 83 (7)	1433 (10) 6483 (47) 5278 (38) 638 (5)	1654 (7) 9595 (43) 10,348 (46) 945 (4)	3292 (9) 16,658 (44) 16,003 (43) 1666 (4)
Symptoms of anxiety/depression,^a^ *N* (%)	145 (12)	1253 (9)	1525 (7)	2923 (8)
COVID‐19 infected before February 22, *N* (%)	255 (20)	3091 (22)	5230 (23)	8576 (23)
SARS‐CoV‐2 vaccine before February 22, *N* (%)	1204 (98)	13,581 (98)	22,213 (98)	36,880 (98)

^a^Hopkins symptom Checklist, HSCL‐5 ≥ 2.

### 3.3 Main Results

We found a significant association between physical activity levels, measured in February 2022, and new bothersome headache. Compared to the moderate/high physically active group, those with low physical activity levels and inactive individuals had increased risk of new bothersome headache (Table 2). After adjusting for covariates, this association persisted with slightly reduced estimates: inactive individuals demonstrated 26% increased risk of experiencing new bothersome headache, while the risk was 22% increased for those with low physical activity levels compared to moderate to high activity levels. Compared to moderate/high physical activity levels we found an increased absolute risk difference of 2.3% and 1.9% for the inactive and the low physical active groups, respectively.

**TABLE 2:** Risk of new bothersome headache by physical activity level: adjusted absolute risks and risk differences and unadjusted and adjusted odds ratios.

	Headache cases^a^ *n* (%)	Absolute risk% (95% CI), adjusted^b^	Absolute risk difference% (95% CI) adjusted^b^	Odds ratio (95% CI), unadjusted	Odds ratio (95% CI), adjusted^b^
Total study sample	4196 (11.1)				
Moderate/high physical active	2256 (10.0)	10.4 (10.0 to 10.8)	Ref.	Ref.	Ref.
Low physical active	1778 (12.8)	12.3 (11.7 to 12.9)	1.9 (1.2 to 2.6)	1.33 (1.25 to 1.42)	1.22 (1.13 to 1.30)
Inactive	172 (13.8)	12.7 (10.8 to 14.6)	2.3 (0.4 to 4.3)	1.45 (1.23 to 1.71)	1.26 (1.05 to 1.51)

^a^Headache frequency in study sample within each category of physical activity.

^b^Adjusted for gender, age, BMI, education, smoking, alcohol and anxiety/depression, COVID‐19 prior to February 22, SARS‐CoV‐2 vaccinated prior to February 22.

OR: odds ratio; AR: absolute risk; ARD: absolute risk difference; RR: relative risk.

### 3.4 SARS‐CoV‐2 Infection and Vaccination

Stratified analysis by COVID‐19 showed that lower levels of physical activity were associated with increased odds of new bothersome headache in both SARS‐CoV‐2 infected and non‐infected participants. These associations remained after adjustment for covariates, although confidence intervals were wider in all groups compared to the unstratified analysis. The corresponding absolute risk differences were 1.9% and 1.3% in the infected participants, and 2.6% and 2.3% in the non‐infected participants, for inactivity and low physical activity, respectively, compared to moderate/high physical activity. The estimate for inactivity among infected participants was imprecise, with confidence intervals including the null.

The distribution of headache across physical activity categories remained consistent in both stratifications, indicating a negligible change in the relationship between new bothersome headache and physical activity levels by the influence of infection status. Further details are provided in Table 3. Additionally, interaction analysis found no significant interaction between COVID‐19 and inactivity (*p* = 0.96) or low physical activity (*p* = 0.31) on new bothersome headache.

**TABLE 3:** Risk of new bothersome headache by physical activity level, stratified by COVID‐19 infection status (February–June 2022): unadjusted and adjusted odds ratios and adjusted absolute risks and risk differences.

		Headache cases^a^ *n* (%)	Absolute risk % (95% CI), adjusted^b^	Absolute risk difference% (95% CI) adjusted^b^	Odds ratio (95% CI) unadjusted	Odds ratio (95% CI) adjusted^b^
COVID‐19 infected	Moderate/high physical activity level	960 (11.0)	11.5 (10.8 to 12.2)	Ref.	Ref.	Ref.
	Low physical activity level	693 (13.7)	12.9 (11.9 to 13.8)	1.3 (0.1 to 2.5)	1.28 (1.16 to 1.43)	1.13 (1.01 to 1.27)
	Physically inactive	68 (15.2)	13.5 (10.3 to 16.7)	1.9 (–1.4 to 5.2)	1.45 (1.11 to 1.89)	1.2 (0.89 to 1.60)
Not infected	Moderate/high physical activity level	1286 (9.3)	9.7 (9.1 to 10.2)	Ref.	Ref.	Ref.
	Low physical activity level	1085 (12.3)	12.0 (11.3 to 12.7)	2.3 (1.5 to 3.2)	1.37 (1.26 to 1.50)	1.28 (1.17 to 1.41)
	Physically inactive	104 (13.0)	12.2 (9.9 to 14.6)	2.6 (0.2 to 5.0)	1.46 (1.18 to 1.81)	1.31 (1.04 to 1.66)

^a^ Headache frequency in study sample within each category of physical activity.

^b^ Adjusted for gender, age, BMI, education, smoking, alcohol and anxiety/depression, COVID‐19 prior to February 22, SARS‐CoV‐2 vaccinated prior to February 22.

OR, odds ratio.

Most participants received SARS‐CoV‐2 vaccines between October 2021 and February 2022, with only a small proportion (2%) vaccinated between February and June 2022. Stratified analysis for this period lacked sufficient power to draw meaningful conclusions due to the limited sample size in each physical activity category by vaccinated participants.

## 4 Discussion

In this study, using a large prospective population‐based cohort, we found a clear association between low physical activity levels and new onset of bothersome headaches. The analyses consistently demonstrated that individuals without prior headache who were inactive or engaged in lower levels of physical activity had a 20%–30% increased risk of new bothersome headache, compared to individuals with moderate to high physical activity levels. These associations persisted after adjusting for potential covariates and were reflected in corresponding absolute risk differences. The results remained consistent across both SARS‐CoV‐2 infected and non‐infected groups, suggesting that the beneficial association between physical activity and reduced risk of new bothersome headache may apply also in the context of exposure to COVID‐19.

There have to our knowledge not been studies investigating the relationship between physical activity and headache either as an acute COVID‐19 symptom or as part of long COVID‐19. However, studies suggest an increased risk of long COVID‐19 symptoms and severe infection for inactive adults (Fernández‐De‐Las‐Peñas et al. 2021). Although our study cannot establish causality, the findings suggest that higher levels of physical activity are associated with a lower likelihood of developing bothersome headache, even in a pandemic context where infection, vaccination, and related factors may increase headache risk.

While SARS‐CoV‐2 vaccines may reduce the risk of infection or lead to milder symptoms, including headache (Mohammed et al. 2022; Notarte et al. 2022), they have also been reported to cause headache as a side effect (Caronna et al. 2023). A multicenter observational study found that most participants experienced worsening of post‐vaccination headache related to physical activity, however, the timing and definition of activity were unclear (Göbel et al. 2021). A recent MoBa study based on the same data material as the present study found that while the first two SARS‐CoV‐2 vaccine doses slightly increased headache risk, COVID‐19 posed a substantially higher risk, and the third dose appeared protective during the high‐transmission Omicron period (Blix et al. 2025). These findings suggest that vaccination may reduce overall headache burden when infection pressure is high. In this context, our findings support physical activity as a complementary, non‐pharmacological strategy to help reduce headache risk in similar periods.

During the pandemic, health services were pressured (Filip et al. 2022). Headache is one of the most frequent reasons for consulting primary healthcare (Finley et al. 2018). Preventative measures to reduce headaches may reduce the need for health care services. Our findings suggest that promoting physical activity may decrease headache occurrence, thus, potentially lowering pressure on healthcare systems. Such measures are of importance, particularly during crises, as they have the potential to contribute to the management of future pandemics.

This is the first prospective cohort study evaluating the influence of physical activity on new bothersome headache in the context of a pandemic. Despite exercise being widely recommended as a preventative measure, few prospective studies have examined whether physical activity is associated with reduced risk of headache. Two studies conducted on another Norwegian cohort have previously investigated physical activity relation to headache risk (Hagen et al. 2018; Varkey et al. 2008). One of these studies identified a dose‐response relationship between physical activity and headache, but with limited adjustment for covariates and the “headache‐free” baseline population defined based on analgesic use (Varkey et al. 2008). Our findings are also consistent with a large prospective cohort study of Swedish men, where lower cardiovascular fitness in early adulthood was associated with a higher long‐term risk of prescription‐requiring migraine (Nyberg et al. 2019). Our study strengthens previous findings (Nyberg et al. 2019; Varkey et al. 2008) with broader adjustment for covariates, and extends the evidence to a pandemic context. While caution must be taken in the interpretation of these findings, it underscores the potential importance of regular physical activity in mitigating the risk of new bothersome headache in the future.

Physical activity level has shown a U‐shaped relationship with other pain conditions such as back pain, where both too little and too much activity equally increases the risk of pain (Heneweer et al. 2009). Similar findings are described for the immune response. While meeting activity recommendations protects against viral infections, excessive exercise can have adverse effects (Scheffer and Latini 2020). Our study could not differentiate between adequate and excessive activity, and to our knowledge such studies have not been done in a headache population. If similar U‐shaped patterns exist in headache populations, the inclusion of individuals with excessive physical activity in the moderate/high activity group could have attenuated the protective association observed for this group. The true protective effect of optimal physical activity may therefore be stronger than estimated in our analysis. Differentiating between adequate and excessive physical activity levels is therefore warranted in future studies.

It is hypothesized that physical activity may prevent headache onset and severity through several physiological mechanisms. A review of observational and experimental studies show that regular physical exercise improves cerebrovascular function, including endothelial health and cerebral blood flow (Amin et al. 2018; Alves et al. 2025), mechanisms also targeted by pharmacological migraine prophylaxis (Amin et al. 2018). Physical exercise can also activate endogenous pain modulation systems by increasing beta‐endorphins and endocannabinoids, enhancing central inhibitory control (Amin et al. 2018; Lima et al. 2017). Additionally, research on chronic pain supports exercise as a key treatment component, offering both physiological and functional benefits (Cohen et al. 2021). These mechanisms may explain how physical activity could reduce headache risk and burden, although further prospective studies are needed to explore these effects and their clinical significance. However, headaches, like several other health conditions, are rarely the result of a single cause but rather influenced by multiple factors (Amiri et al. 2022; Nicholas 2022; Rothman 2012). Conditions such as anxiety, depression, sleep disturbances, and BMI are associated with headache (Agbetou and Adoukonou 2022; Amiri et al. 2022; Holroyd et al. 2000), but also inactivity (Lima et al. 2017). Physical activity can potentially improve mental health, sleep quality and lower BMI levels (Lima et al. 2017). Our findings may be explained by the role of physical activity in mitigating other risk factors for bothersome headache.

### 4.1 Strengths and Limitations

This study benefits from a large, nationwide sample, enhancing statistical power and allowing adjustment for relevant covariates. Importantly, participants were not recruited to investigate headache, which reduces the risk of self‐selection bias. Physical activity was assessed using two validated self‐report items (Kurtze et al. 2007; Nes et al. 2011) addressing frequency and intensity of activity. However, the dataset lacked information on duration which limits precision in classifying activity levels (Kurtze et al. 2007; Nes et al. 2011; World Health Organization 2020), potentially leading to overestimation of moderate/high activity levels, thus lower estimates. Physical activity data were collected in February 2022, after most COVID‐19 restrictions in Norway had been lifted, providing relatively stable conditions (Den norske regjeringen Hoo 2023). Although activity parameters were collected at a single time point, the measure likely reflects typical activity patterns in the preceding months. Timely data collection has been essential for understanding the relationship between physical activity and headache during the COVID‐19 pandemic, when activity levels were likely affected by social restrictions (Stockwell et al. 2021).

Although several covariates were adjusted for, unmeasured confounding such as comorbid pain conditions or life stressors cannot be ruled out. Information on headache‐related medication use was not available in the dataset and could therefore not be included in the analyses. The smoking variable emphasized current smoking behaviour, including occasional smoking, which may limit comparability with studies conducted in settings where smoking patterns differ. However, the broad set of available variables likely reduced residual bias. The baseline headache data were collected 1.5 years into the pandemic, possibly excluding cases related to COVID‐19 or SARS‐CoV‐2 vaccination. Yet, few participants had contracted the virus before baseline, suggesting limited impact. Due to the widespread SARS‐CoV‐2 vaccination in the cohort prior to study start, isolating vaccine effects was not possible. Additionally, while headache diagnosis was available, subgroup analyses by headache type were not feasible due to limited power.

Individuals with higher socioeconomic status are overrepresented in MoBa (Biele et al. 2019) and this study did not include younger or childless adults. Incident headache is more common among younger than middle aged individuals (Stovner et al. 2022). Additionally, the high SARS‐CoV‐2 vaccination coverage within the cohort may limit generalizability to settings with lower coverage. Taking these limitations into account, the large sample size, prospective design, and availability of key covariates strengthen the validity and generalizability of the findings. Future studies should aim to include younger populations and explore additional sources of potential confounding to better understand the relationship between physical activity and headache.

## 5 Conclusion

Our findings highlight a robust association between physical activity levels and new bothersome headache, unaffected by COVID‐19. Promoting regular physical activity may serve as a low‐cost, accessible strategy to reduce headache risk, particularly during periods of widespread lifestyle disruption such as pandemics.

Further research is needed to understand underlying mechanisms and optimize interventions aimed at reducing headache burden in the population. Investigating modifiable potential risk reducing factors like physical activity is important, particularly during pandemics, yet research in this area remains limited. Further studies should try to separate higher levels of physical activity for further insight in specific recommendations.

### 5.1 Study Highlights

- The study showed significant association between lower physical activity levels and new bothersome headache during the pandemic.
- This association was consistent across COVID‐19 status.
- Despite certain demographic limitations in the study sample, regular physical activity potentially mitigates the risk of bothersome headache.
- The findings suggest that promoting physical activity could serve as a preventive strategy for headache management.

## Author Contributions

**Anne‐Mari Torgersen Dalgeir**: Conceptualization, methodology, data curation, validation, formal analysis, visualization, project administration, resources, writing – original draft, writing – review & editing. **Ida Henriette Caspersen**: Conceptualization, methodology, data curation, investigation, validation, formal analysis, supervision, visualization, project administration, resources, writing – original draft, writing – review & editing. **Edoardo Caronna**: Conceptualization, validation, writing – review & editing. **Per Magnus**: Conceptualization, data curation, investigation, funding acquisition, resources, writing – review & editing. **Lill Trogstad**: Data curation, funding acquisition, resources, writing – review & editing. **Marte‐Helene Bjørk**: Conceptualization, methodology, validation, supervision, visualization, project administration, resources, writing – original draft, writing – review & editing.

## Funding

The MoBa project received funding from various sources including the Norwegian Ministry of Health, the Norwegian Research Council and the Confederation of Norwegian Business and Industry. IHC and PM was funded by the Norwegian Research Council's Centres of Excellence Funding Scheme (no. 262700).

## Conflicts of Interest

Marte‐Helene Bjørk has the last 3 years received advisory board honoraria and/or speaking honoraria from marked authorization holders of migraine preventive drugs (Novartis, Pfizer, AbbVie, Organon, and Lundbeck).

Edoardo Caronna has received honoraria from Novartis, Chiesi, Lundbeck, MedScape, Lilly; his salary has been partially funded by Río Hortega grant Acción Estratégica en Salud 2017–2020 from Instituto de Salud Carlos III (CM20/00217) and Juan Rodés fellowship, Subprograma Estatal de Incorporación de la Acción Estratégica en Salud 2023 (JR23/00065). He is a junior editor for Cephalalgia.

## Data Availability

Data from the Norwegian Mother, Father and Child Cohort Study used in this study are managed by the national health register holders in Norway (Norwegian Institute of Public Health) and can be made available to researchers, provided approval from the Regional Committees for Medical and Health Research Ethics, compliance with the EU General Data Protection Regulation and approval from the data owners. The consent given by the participants does not open for storage of data on an individual level in repositories or journals. Researchers who want access to data sets for replication should apply through helsedata.no. Access to data sets requires approval from The Regional Committee for Medical and Health Research Ethics in Norway and an agreement with MoBa.

## References

[brb371490-bib-0031] Agbetou, M. , and T. Adoukonou . 2022. “Lifestyle Modifications for Migraine Management.” Frontiers in Neurology 13: 719467. 35370920 10.3389/fneur.2022.719467 PMC8971279

[brb371490-bib-0026] Alves, L. , D. Hashiguchi , C. M. Loss , et al. 2025. “Vascular Dysfunction in Alzheimer's Disease: Exploring the Potential of Aerobic and Resistance Exercises as Therapeutic Strategies.” Journal of Alzheimer's Disease 104: 963–979. 10.1177/13872877251321118 40079781

[brb371490-bib-0004] Amin, F. M. , S. Aristeidou , C. Baraldi , et al. 2018. “The Association Between Migraine and Physical Exercise.” The Journal of Headache and Pain 19: 1–9. 30203180 10.1186/s10194-018-0902-y PMC6134860

[brb371490-bib-0002] Amiri, P. , S. Kazeminasab , S. A. Nejadghaderi , et al. 2022. “Migraine: A Review on Its History, Global Epidemiology, Risk Factors, and Comorbidities.” Frontiers in Neurology 12: 800605. 35281991 10.3389/fneur.2021.800605 PMC8904749

[brb371490-bib-0034] Biele, G. , K. Gustavson , N. O. Czajkowski , et al. 2019. “Bias From Self Selection and Loss to Follow‐Up in Prospective Cohort Studies.” European Journal of Epidemiology 34: 927–938. 31451995 10.1007/s10654-019-00550-1

[brb371490-bib-0018] Blix, K. , I. Laake , I. H. Caspersen , et al. 2025. “New‐Onset Headache After SARS‐CoV‐2 Infection and Vaccination: Results From Three Population‐Based Cohorts in Norway.” European Journal of Neurology 32, no. 12: e70437. 41340357 10.1111/ene.70437 PMC12675824

[brb371490-bib-0006] Caronna, E. , T. C. Van Den Hoek , H. Bolay , et al. 2023. “Headache Attributed to SARS‐CoV‐2 Infection, Vaccination and the Impact on Primary Headache Disorders of the COVID‐19 Pandemic: A Comprehensive Review.” Cephalalgia 43: 03331024221131337. 10.1177/03331024221131337 36606562

[brb371490-bib-0028] Cohen, S. P. , L. Vase , and W. M. Hooten . 2021. “Chronic Pain: An Update on Burden, Best Practices, and New Advances.” The Lancet 397: 2082–2097. 10.1016/S0140-6736(21)00393-7 34062143

[brb371490-bib-0032] Den norske regjeringen Hoo . 2023. *Tidslinje: Myndighetenes Håndtering av Koronasituasjonen*. Regjeringen.no.

[brb371490-bib-0005] Fernández‐De‐Las‐Peñas, C. , M. Navarro‐Santana , V. Gómez‐Mayordomo , et al. 2021. “Headache as an Acute and Post‐COVID‐19 Symptom in COVID‐19 Survivors: A Meta‐Analysis of the Current Literature.” European Journal of Neurology 28: 3820–3825. 34327787 10.1111/ene.15040 PMC8444899

[brb371490-bib-0019] Filip, R. , R. Gheorghita Puscaselu , L. Anchidin‐Norocel , M. Dimian , and W. K. Savage . 2022. “Global Challenges to Public Health Care Systems During the COVID‐19 Pandemic: A Review of Pandemic Measures and Problems.” Journal of Personalized Medicine 12: 1295. 36013244 10.3390/jpm12081295 PMC9409667

[brb371490-bib-0020] Finley, C. R. , D. S. Chan , S. Garrison , et al. 2018. “What Are the Most Common Conditions in Primary Care?: Systematic Review.” Canadian Family Physician 64: 832–840. 30429181 PMC6234945

[brb371490-bib-0010] Folkehelseinstituttet . 2023. “MoBa‐Spørreundersøkelse om Koronavirus.” https://www.fhi.no/op/studier/moba/undersokelser/moba‐undersokelse‐om‐koronavirus/.

[brb371490-bib-0007] Göbel, C. H. , A. Heinze , S. Karstedt , et al. 2021. “Clinical Characteristics of Headache After Vaccination Against COVID‐19 (Coronavirus SARS‐CoV‐2) With the BNT162b2 mRNA Vaccine: A Multicentre Observational Cohort Study.” Brain Communications 3: fcab169. 34405142 10.1093/braincomms/fcab169 PMC8344581

[brb371490-bib-0011] Hagen, K. , J.‐A. Zwart , L. Vatten , L.j Stovner , and G. Bovim . 2000. “Head‐HUNT: Validity and Reliability of a Headache Questionnaire in a Large Population‐Based Study in Norway.” Cephalalgia 20: 244–251. 10999674 10.1046/j.1468-2982.2000.00049.x

[brb371490-bib-0021] Hagen, K. , A. N. Åsberg , L. Stovner , et al. 2018. “Lifestyle Factors and Risk of Migraine and Tension‐Type Headache. Follow‐Up Data From the Nord‐Trøndelag Health Surveys 1995–1997 and 2006–2008.” Cephalalgia 38: 1919–1926. 29517305 10.1177/0333102418764888

[brb371490-bib-0024] Heneweer, H. , L. Vanhees , and H. S. J. Picavet . 2009. “Physical Activity and Low Back Pain: A U‐Shaped Relation?” Pain 143: 21–25. 19217208 10.1016/j.pain.2008.12.033

[brb371490-bib-0003] Holroyd, K. A. , M. Stensland , G. L. Lipchik , K. R. Hill , F. S. O'Donnell , and G. Cordingley . 2000. “Psychosocial Correlates and Impact of Chronic Tension‐Type Headaches.” Headache: The Journal of Head and Face Pain 40: 3–16. 10.1046/j.1526-4610.2000.00001.x PMC2128255 10759896

[brb371490-bib-0001] James, S. L. , D. Abate , K. H. Abate , et al. 2018. “Global, Regional, and National Incidence, Prevalence, and Years Lived With Disability for 354 Diseases and Injuries for 195 Countries and Territories, 1990–2017: A Systematic Analysis for the Global Burden of Disease Study 2017.” The Lancet 392: 1789–1858. 10.1016/S0140-6736(18)32279-7. PMC6227754 30496104

[brb371490-bib-0008] Jimeno‐Almazán, A. , A. Martínez‐Cava , Á. Buendía‐Romero , et al. 2022. “Relationship Between the Severity of Persistent Symptoms, Physical Fitness, and Cardiopulmonary Function in Post‐COVID‐19 Condition. A Population‐Based Analysis.” Internal and Emergency Medicine 17: 2199–2208. 35904700 10.1007/s11739-022-03039-0 PMC9335466

[brb371490-bib-0012] Kurtze, N. , V. Rangul , B.‐E. Hustvedt , and W. D. Flanders . 2007. “Reliability and Validity of Self‐Reported Physical Activity in the Nord‐Trøndelag Health Study (HUNT 2).” European Journal of Epidemiology 22: 379–387. 17356925 10.1007/s10654-007-9110-9

[brb371490-bib-0027] Lima, L. V. , T. S. Abner , and K. A. Sluka . 2017. “Does Exercise Increase or Decrease Pain? Central Mechanisms Underlying These Two Phenomena.” The Journal of Physiology 595: 4141–4150. 28369946 10.1113/JP273355 PMC5491894

[brb371490-bib-0009] Magnus, P. , C. Birke , K. Vejrup , et al. 2016. “Cohort Profile Update: The Norwegian Mother and Child Cohort Study (MoBa).” International Journal of Epidemiology 45: 382–388. 27063603 10.1093/ije/dyw029

[brb371490-bib-0017] Mohammed, I. , A. Nauman , P. Paul , et al. 2022. “The Efficacy and Effectiveness of the COVID‐19 Vaccines in Reducing Infection, Severity, Hospitalization, and Mortality: A Systematic Review.” Human Vaccines & Immunotherapeutics 18: 2027160. 35113777 10.1080/21645515.2022.2027160 PMC8862168

[brb371490-bib-0013] Nes, B. M. , I. Janszky , L. J. Vatten , T. I. L. Nilsen , S. T. Aspenes , and U. Wisløff . 2011. “Estimating V·_O2_peak From a Nonexercise Prediction Model: The HUNT Study, Norway.” Medicine & Science in Sports & Exercise 43: 2024–2030. 21502897 10.1249/MSS.0b013e31821d3f6f

[brb371490-bib-0029] Nicholas, M. K. 2022. “The Biopsychosocial Model of Pain 40 Years On: Time for a Reappraisal?” Pain 10: 1097. 10.1097/j.pain.0000000000002654 36252231

[brb371490-bib-0016] Notarte, K. I. , J. A. Catahay , J. V. Velasco , et al. 2022. “Impact of COVID‐19 Vaccination on the Risk of Developing Long‐COVID and on Existing Long‐COVID Symptoms: A Systematic Review.” EClinicalMedicine 53: 101624. 36051247 10.1016/j.eclinm.2022.101624 PMC9417563

[brb371490-bib-0023] Nyberg, J. , S. Gustavsson , M. Linde , et al. 2019. “Cardiovascular Fitness and Risk of Migraine: A Large, Prospective Population‐Based Study of Swedish Young Adult Men.” BMJ Open 9: e029147. 10.1136/bmjopen-2019-029147 PMC6719773 31473616

[brb371490-bib-0030] Rothman, K. J. 2012. Epidemiology: An Introduction. 2 ed. New York: Oxford University Press.

[brb371490-bib-0025] Scheffer, D. a. L. , and A. Latini . 2020. “Exercise‐Induced Immune System Response: Anti‐Inflammatory Status on Peripheral and central Organs.” Biochimica et Biophysica Acta (BBA)—Molecular Basis of Disease 1866: 165823. 32360589 10.1016/j.bbadis.2020.165823 PMC7188661

[brb371490-bib-0033] Stockwell, S. , M. Trott , M. Tully , et al. 2021. “Changes in Physical Activity and Sedentary Behaviours From Before to During the COVID‐19 Pandemic Lockdown: A Systematic Review.” BMJ Open Sport & Exercise Medicine 7: e000960. 10.1136/bmjsem-2020-000960 PMC7852071 34192010

[brb371490-bib-0035] Stovner, L. J. , K. Hagen , M. Linde , and T. J. Steiner . 2022. “The Global Prevalence of Headache: An Update, With Analysis of the Influences of Methodological Factors on Prevalence Estimates.” The Journal of Headache and Pain 23: 34. 35410119 10.1186/s10194-022-01402-2 PMC9004186

[brb371490-bib-0015] Strand, B. H. , O. S. Dalgard , K. Tambs , and M. Rognerud . 2003. “Measuring the Mental Health Status of the Norwegian Population: A Comparison of the Instruments SCL‐25, SCL‐10, SCL‐5 and MHI‐5 (SF‐36).” Nordic Journal of Psychiatry 57: 113–118. 12745773 10.1080/08039480310000932

[brb371490-bib-0022] Varkey, E. , K. Hagen , J. Zwart , and M. Linde . 2008. “Physical Activity and Headache: Results From the Nord‐Trøndelag Health Study (HUNT).” Cephalalgia 28: 1292–1297. 18771495 10.1111/j.1468-2982.2008.01678.x

[brb371490-bib-0014] World Health Organization . 2020. “WHO Guidelines on Physical Activity and Sedentary Behaviour.” WHO. 10.1136/bjsports-2020-102955 PMC7719906 33239350

